# High throughput screening of mitochondrial bioenergetics in human differentiated myotubes identifies novel enhancers of muscle performance in aged mice

**DOI:** 10.1038/s41598-018-27614-8

**Published:** 2018-06-20

**Authors:** Nadine Biesemann, Janina S. Ried, Danping Ding-Pfennigdorff, Axel Dietrich, Christine Rudolph, Steffen Hahn, Wolfgang Hennerici, Christian Asbrand, Thomas Leeuw, Carsten Strübing

**Affiliations:** 1 R&D Immunology & Inflammation Therapeutic Area, Sanofi, Industriepark Hoechst, 65926 Frankfurt am Main, Germany; 2 R&D TMED Bioinformatics, Sanofi, Industriepark Hoechst, 65926 Frankfurt am Main, Germany; 3 R&D Integrated Drug Discovery, Sanofi, Industriepark Hoechst, 65926 Frankfurt am Main, Germany; 4 R&D Clinical Science Operations, Sanofi, Industriepark Hoechst, 65926 Frankfurt am Main, Germany

## Abstract

Mitochondrial dysfunction is increasingly recognized as a contributor to age-related muscle loss and functional impairment. Therefore, we developed a high throughput screening strategy that enabled the identification of compounds boosting mitochondrial energy production in a human skeletal muscle cell model. Screening of 7949 pure natural products revealed 22 molecules that significantly increased oxygen consumption and ATP levels in myotubes. One of the most potent compounds was the flavanone hesperetin. Hesperetin (10 µM) increased intracellular ATP by 33% and mitochondrial spare capacity by 25%. Furthermore, the compound reduced oxidative stress in primary myotubes as well as muscle tissue *in vivo*. In aged mice administration of hesperetin (50 mg/kg/d) completely reverted the age-related decrease of muscle fiber size and improved running performance of treated animals. These results provide a novel screening platform for the discovery of drugs that can improve skeletal muscle function in patients suffering from sarcopenia or other disorders associated with mitochondrial dysfunction.

## Introduction

Sarcopenia, the age-dependent loss of skeletal muscle mass and function is associated with increased mortality and morbidity in affected patients and presents an increasing burden to aging societies. Physical exercise is the only intervention shown to prevent and decrease sarcopenia but pharmacological treatment options are not yet available^1^.

Although the molecular pathways contributing to sarcopenia and accelerated decrease of muscle performance in elderly are only incompletely understood, mitochondrial dysfunction and aberrant bioenergetics are likely to play an important role in the pathophysiology. In particular, downregulation of mitochondrial biogenesis, oxygen consumption and ATP production have been observed in physically inactive elderly as well as older subjects with low physical performance^2–4^, which is one of the clinical criteria defining severe sarcopenia^5^. Likewise, in animal models such as sarcopenic rats a prominent downregulation of genes involved in oxidative phosphorylation and mitochondrial metabolism was observed that correlated more closely with muscle loss than with age per se^6^. Furthermore, aging rats and mice with increased mitochondrial mutations that develop sarcopenia show a decrease in total mitochondrial ATP content^7,8^, linking mitochondrial dysfunction with ATP content. Aging rodents also show functional impairment of mitochondrial energetics at physiological relevant levels of muscle activity^9^, indicating that animal models can be used to study therapeutic interventions targeting mitochondria. Indeed, treatment of old mice with SS-31, a mitochondria-targeted peptide that reduces reactive oxygen species (ROS) generation and enhances ATP production^10^ led to significant higher fatigue resistance and muscle endurance^11^. Thus, despite the as yet uncertain causality between mitochondrial dysfunction, reduced mobility, and muscle wasting, compounds that improve oxidative metabolism may have therapeutic benefit in patients with sarcopenia and other disorders associated with abnormal cellular respiration.

Due to the complexity of mitochondria and the multitude of regulatory mechanisms governing organelle function, identification of positive modulators has proven difficult. Recently however, several groups have developed integrated screening strategies using phenotypic, biochemical and whole animal assays that facilitated the discovery of novel mitochondrial modulators^12^. We adopted the multidimensional screening approach to select compounds that increased mitochondrial ATP in differentiated human myotubes. Screening of a large natural product collection on this highly relevant, postmitotic cell model identified novel enhancers of skeletal muscle respiration including the flavanone hesperetin. *In vitro* effects of hesperetin translated into improved physical performance of frail mice. Thus, our experiments establish a robust assay platform that will facilitate the discovery of mitochondria-targeted drugs for the treatment of age-related muscle disorders.

## Results

### Multi-parameter high throughput screening for mitochondrial activators in human differentiated myotubes

**Figure 1:**
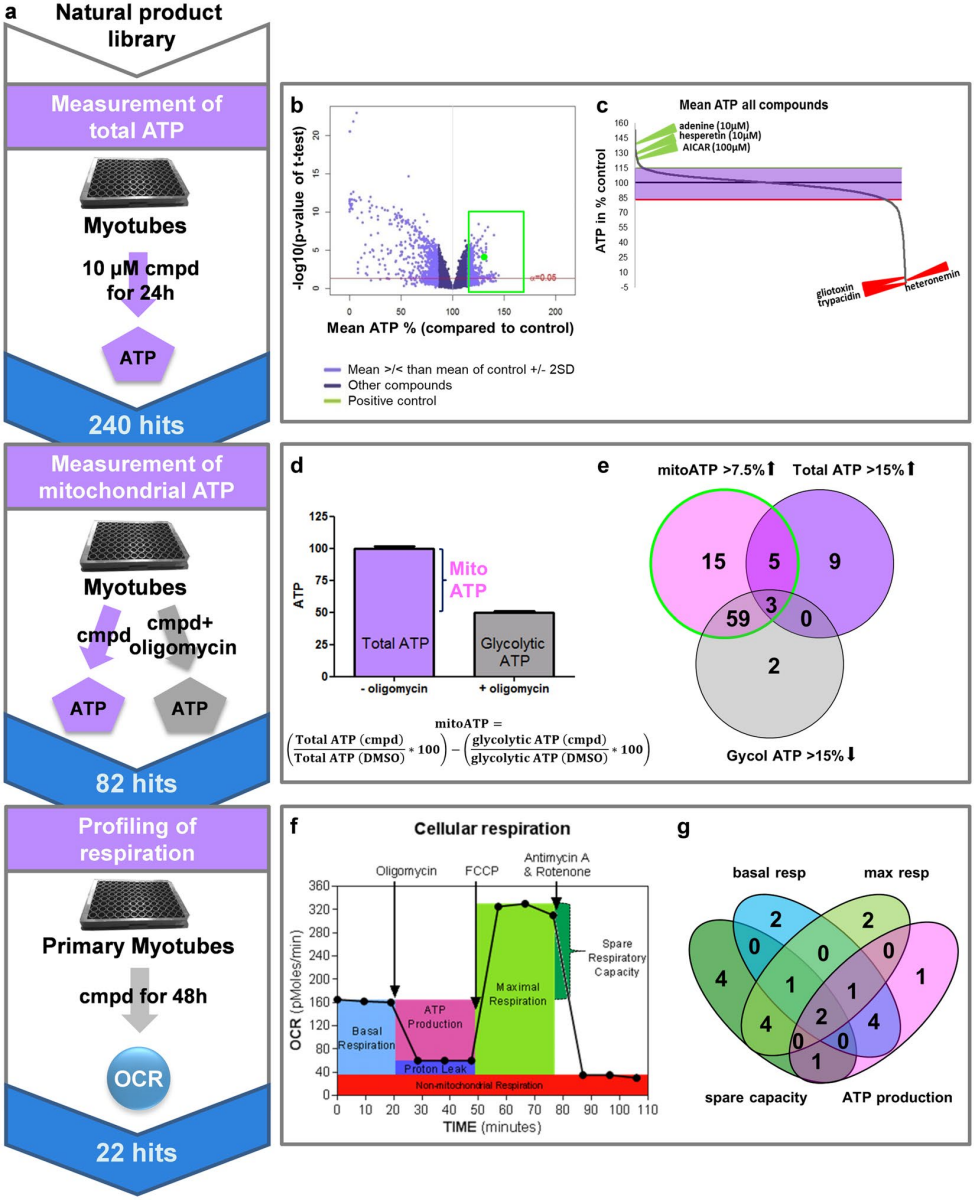
Multi-dimensional screen for mitochondrial activators. (**a**) Screening strategy: Primary screening of Sanofi’s pure natural compound library was conducted in differentiated immortalized human myotubes by measuring total ATP content after compound treatment in low glucose medium. 240 primary hits were further examined in a dual ATP assay with and without the oxidative phosphorylation inhibitor oligomycin to assess compound effects on mitochondrial and glycolytic ATP content. Finally, mitochondrial effects of active compounds were verified by direct measurement of cellular respiration in differentiated primary human myotubes. (**b**) Volcano plot illustrating nominal significance versus change of mean relative ATP content for all 7949 compounds screened. Standard deviation of data was 7.5% leading to the identification of 240 primary hits (shown in green box) which increased ATP by ≥2 SD. (**c**) Dot plot of mean ATP in % of control for all tested compounds. Selected active and toxic compounds are highlighted in green and red respectively. (**d**) Schematic representation of determination of mitochondrial, glycolytic and total ATP content from experimental data. For each compound cellular ATP levels were measured in untreated and oligomycin-treated myotubes (n ≥ 4); the difference was considered as mitochondrial ATP. (**e**) Venn diagram illustrating compound sets with combinatorial effects on mitochondrial, total and glycolytic ATP. Number of compounds in each category is given. The green circle marks the selected hit set defined by significantly increasing total ATP or decreasing glycolytic ATP and by increasing mitochondrial ATP level more than 7.5%. (**f**) Schematic overview of cellular respiration profiling using Seahorse XFe96 analyzer. Primary differentiated myotubes were treated 48 h with compounds or DMSO and cellular respiration was analyzed in real-time. Different components of mitochondrial respiration were isolated by treating myotubes with oligomycin, FCCP and antimycin/rotenone. Compounds that increased one or several components of mitochondrial respiration significantly were considered as hits. (**g**) Venn diagram highlighting the distribution of hits with differential effects on basal respiration, spare capacity, maximal respiration or ATP production (n ≥ 8, 1–3 independent experiments). OCR: oxygen consumption rate.

To identify positive modulators of oxidative phosphorylation in skeletal muscle, we developed a systematic phenotypic screening pipeline using differentiated human myotubes (Fig. 1a). Our screening cascade included three assays – 1. An ATP assay that was employed for primary screening, 2. a secondary ATP assay that allowed to differentiate between glycolysis-derived and mitochondrial ATP production, and 3. real-time respirometry (Seahorse) to directly assess mitochondrial function. For high throughput measurements of cellular ATP myotubes differentiated from an immortalized myoblast line (hSkMc) were used whereas respiratory measurements were done on primary differentiated myotubes. This screening strategy led to the identification of 22 active compounds that improved mitochondrial function in primary myotubes (Fig. 1a).

Primary screening was performed by treating immortalized differentiated myotubes with 10 µM pure natural products for 24 h. 1200 compounds (out of ~8000) induced nominal significant increases in ATP levels (Fig. 1b, green box) compared to DMSO-treated controls highlighting the sensitivity of the screen. We defined compounds that increased ATP levels by more than 15% (>2 standard deviation (SD) of DMSO-treated controls) as primary hits. By this definition we observed a hit rate of 3% (240 compounds) and showed that 88% of the compounds did not modulate cellular ATP content. Positives included 62 compounds that increased intracellular ATP more strongly compared to our positive control the AMPK activator 5-Aminoimidazole-4-carboxamide ribonucleotide (AICAR) even though AICAR was used at a 10-fold higher concentration (100 µM) (Fig. 1b,c). Adenine and hesperetin, an antioxidant from citrus peels^13^, were among the most potent treatments, whereas known toxic compounds like gliotaxin, trypacidin, and heteronemin significantly decreased ATP content (Fig. 1c).

Next, we sought to determine whether changes in ATP content were linked to mitochondrial respiration. To that end mitochondrial ATP was analyzed in myotubes grown for 3 h in galactose to suppress glycolysis. The difference between ATP content in the presence and absence of oligomycin, an inhibitor of oxidative phosphorylation, was considered to reflect mitochondrial contribution to total ATP (Fig. 1d). Evaluation of compound effects under these conditions showed that 82 substances (from the 240 hits) increased ATP content specifically via oxidative phosphorylation (Fig. 1a,e). Furthermore, the majority of compounds increased mitochondrial ATP, while simultaneously decreasing glycolytic ATP (Fig. 1e). To prove directly that active compounds upregulated mitochondrial energy generation we used real-time respirometry to monitor cellular oxygen consumption. These experiments were performed in primary differentiated myotubes in order to validate our findings in a second physiological cell model (Fig. 1a,f). Quantification of oxygen consumption rate identified 22 final hits with significant effects on one or several parameters of mitochondrial respiration (Fig. 1g). Most hits increased spare respiratory capacity but we also identified compounds that acted on ATP production or basal respiration (Fig. 1g), highlighting the ability of our approach to capture compounds with diverse modes of action.

**Figure 2:**
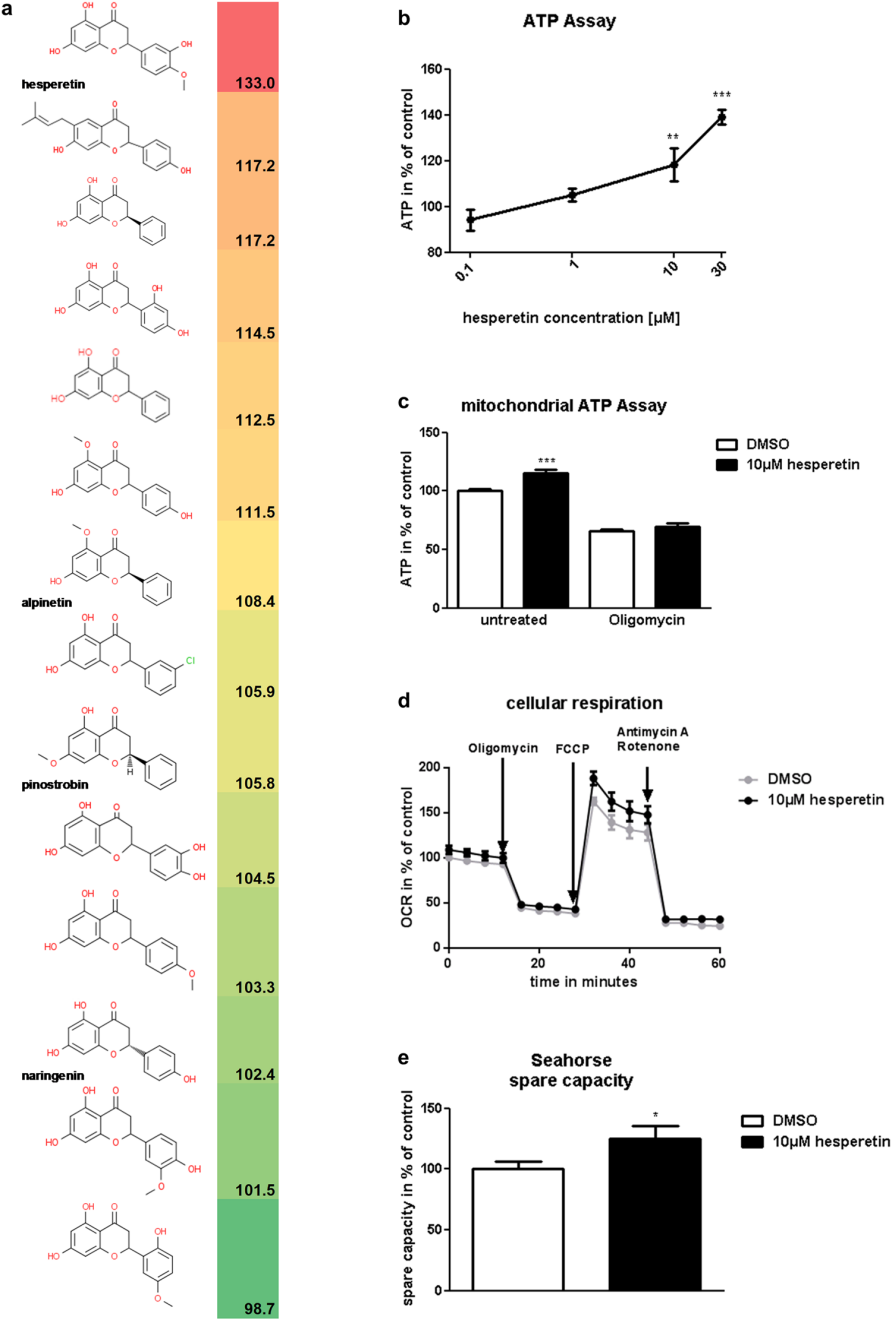
Hesperetin increases mitochondrial ATP content and improves respiration. (**a**) Chemical structures and activities of flavanone series compounds. Activities represent mean cellular ATP in % of control as determined during primary screening in differentiated immortalized human myotubes. (**b**) Dose-dependent stimulation of intracellular ATP by hesperetin. ATP content was measured after 24 h incubation of differentiated immortalized human myotubes with hesperetin or DMSO (control) in low glucose medium (n = 4). (**c**) Hesperetin upregulates mitochondrial ATP content. ATP was measured in differentiated immortalized human myotubes after 21 h incubation in low glucose medium, followed by 3 h in galactose medium with and without 3 µM oligomycin in the continuous presence of hesperetin or DMSO (control) (n ≥ 8). The difference in ATP content between untreated and oligomycin-treated cells was considered as mitochondrial ATP. (**d**) Real-time recording of cellular respiration in primary differentiated myotubes treated for 48 h with 10 µM hesperetin or DMSO (control). Oxygen consumption rate (OCR) was monitored using Seahorse XFe96 analyzer (shown are mean values of 5 independent experiments). Oligomycin, FCCP and antimycin/rotenone were applied to isolate respiration coupled to ATP production, mitochondrial spare capacity and non-mitochondrial respiration. (**e**) Comparison of spare respiratory capacity in hesperetin and DMSO-treated myotubes (n = 31 (hesperetin), n = 72 (DMSO); 5 independent experiments). Spare capacity was calculated from the Seahorse recordings as illustrated in Fig. 1f. Data represent means ± SEM. *P < 0.05; **P < 0.01; ***P < 0.001 ((**b**) One-Way Anova with Dunnett’s test; (**c**,**e**) two-tailed t-test).

Chemical clustering of hit compounds revealed several series of structurally related molecules indicating that mitochondria-stimulating activity was non-randomly associated with certain structural features. The flavanone series shown in Fig. 2a was identified as one of the most promising clusters. This series included hesperetin as well as 2 other compounds that significantly elevated ATP levels by more than 15% in the primary screen (Fig. 2a). A quantitative comparison of compound activities based on dose-response relationships confirmed hesperetin as one of the most effective natural products tested (Fig. 2b). Moreover, evaluation of mitochondria-derived ATP (Fig. 2c) and direct measurements of cellular respiration (Fig. 2d) verified its mitochondria-targeted mechanism of action. In the latter experiments hesperetin specifically increased spare capacity (Fig. 2d,e) which is considered as an indicator of mitochondrial fitness/flexibility^14–16^ and a potential blood biomarker for physical ability that correlated with gait speed in older adults^17^.

### Hesperetin activates *Pgc-1alpha* and *Nrf2 in vitro*

**Figure 3:**
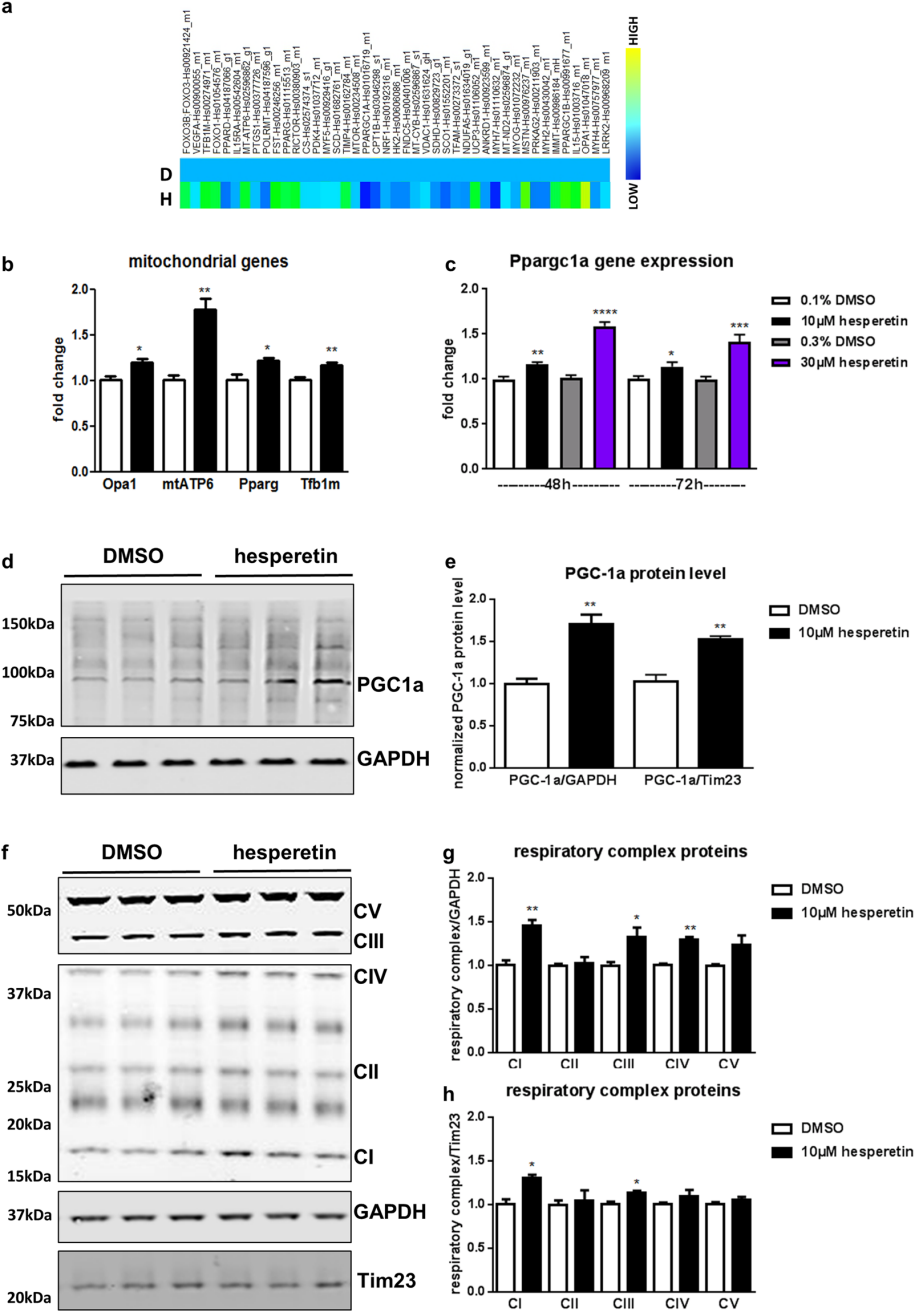
Hesperetin induces mitochondrial biogenesis *in vitro*. (**a**–**h**) Primary differentiated myotubes were treated 48 h with hesperetin or DMSO. (**a**) Custom-made microfluidic gene expression array with mitochondrial and myogenic genes (n = 2). Hesperetin-treated primary differentiated myotubes are marked with H, DMSO control with D. (**b**) Taqman verification of mitochondrial target genes (n = 4). (**c**) Taqman verification of Pgc-1alpha gene expression (Ppargc1a) after 48 and 72 h hesperetin stimulation. (**d**–**h**) Western Blot analysis and quantification of PGC-1alpha (**d**,**e**) and respiratory complexes (**f**–**h**). GAPDH served as loading control and was detected with another fluorescent IRDye (representative blots of n = 3 independent experiments). Shown are cropped gels. Tim23 was used to normalize protein levels to mitochondrial content (**e**,**h**). (**f**) Due to higher protein levels of complex V and III, exposure times were reduced for visualization compared to lower expressed respiratory complexes (I, II and IV). Combined blots are shown in Suppl. Fig. S1. Values represent means ± SEM. *P < 0.05; **P < 0.01; ***P < 0.001; ****P < 0.0001 (two-tailed t-test).

As detailed above, hesperetin emerged from our screen as a potent enhancer of mitochondrial respiration and ATP content in myotubes making it an interesting candidate for further profiling. Although pleiotropic effects, e.g. antioxidant and anti-inflammatory responses, have been assigned to hesperetin^13,18^ its molecular mechanism of action on skeletal muscle is largely unknown. Phenotypically, hesperetin treatment did not affect differentiation status or cell proliferation in already differentiated primary myotubes (Suppl. Fig. 1a,b). To further elucidate the mode of action of the compound, we analyzed expression of myogenic and mitochondrial genes with a custom-made microfluidic card (Fig. 3a) and verified regulation of target genes by Taqman analysis (Fig. 3b). Interestingly, mitochondrial ATP synthase 6 (*Mtatp6)* was highest upregulated after hesperetin stimulation (Fig. 3b). In parallel, mitochondrial regulators like the methyl transferase Tfb1m (transcription factor B1, mitochondrial) were also upregulated. Tfb1m has no transcriptional activity but supports proper mitochondrial translation and metabolism via 12S rRNA methylation^19^.

An important transcriptional master regulator of mitochondrial biogenesis is the coactivator Pgc-1alpha which increases mitochondrial biogenesis and improves oxidative capacity in differentiated myotubes^20^. Pgc-1alpha exerts its effects by directly upregulating transcriptional activity and/or increasing expression of key drivers of mitochondrial gene expression^20^ which in turn stimulate expression of mitochondria-specific proteins including Tfb1m^21^. Although Pgc-1alpha expression was not increased according to our initial analysis (Fig. 3a), follow-up Taqman analysis at different timepoints and concentrations indeed revealed a slight upregulation of Pgc-1alpha gene expression after hesperetin stimulation (Fig. 3c). Furthermore, hesperetin treatment also increased Pgc-1alpha protein levels in differentiated myotubes (Fig. 3d,e).

In line with Pgc-1alpha upregulation we detected increased protein levels of the respiratory chain complexes (Fig. 3f,g, Suppl. Fig. 1c). Notably, all complexes except complex II were upregulated. As all complex II subunits are encoded in the nucleus in contrast to the other respiratory complexes which contain mitochondria-encoded proteins, this may point to the specific regulation of mitochondrial gene expression. Normalizing the protein levels to a mitochondrial inner membrane protein (Tim23) slightly reduced the relative increase in respiratory chain complexes (Fig. 3h) reiterating that mitochondrial proteins were preferentially upregulated.

**Figure 4:**
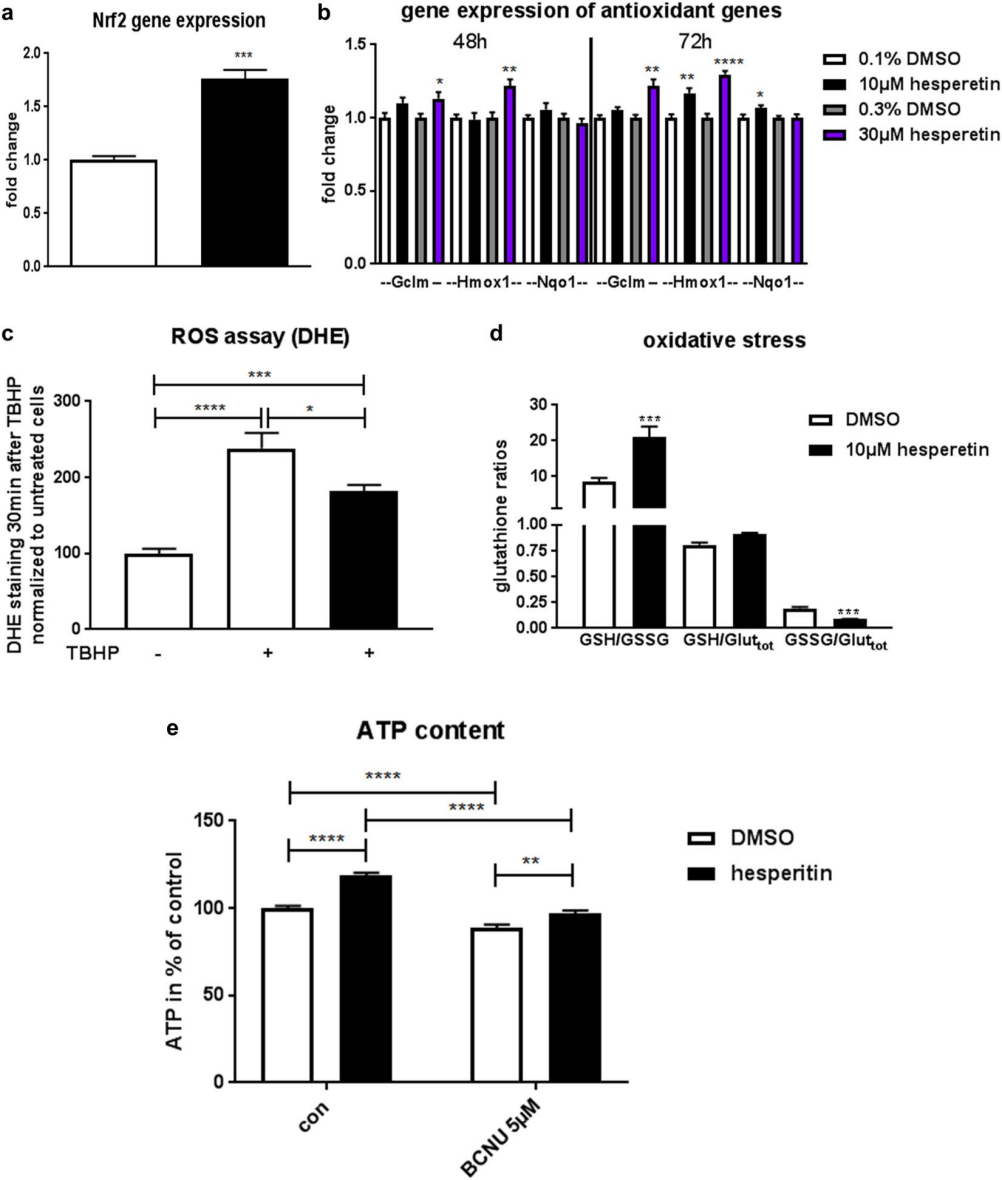
Hesperetin activates Nrf2 and reduces oxidative stress. (**a**–**d**) Primary differentiated myotubes were treated with hesperetin or DMSO. (**a**) Taqman analysis of Nrf2 (gene symbol Nfe2l2) gene expression (n = 4)). (**b**) Taqman analysis of Nrf2-ARE target genes (n = 6). (**c**) DHE live cell imaging of oxidative stress 30 min after stimulation with 100 µM TBHP. Images were taken with ImageXPress and % positive DHE area was analyzed (2 independent experiments, n = 8). (**d**) Measurement of reduced (GSH) and oxidized (GSSG) forms of glutathione in myotubes treated with 150 µM TBHP for 1 h (n = 6). (**e**) ATP content was measured after 24 h incubation of differentiated immortalized human myotubes with hesperetin or DMSO (control) with or without BCNU, an inhibitor of glutathione reductase activity, in low glucose medium (3 independent experiments, n ≥ 24). Values represent means ± SEM. *P < 0.05; **P < 0.01; ***P < 0.001; ****P < 0.0001 (two-tailed t-test (**a**–**c**), one-Way Anova with Dunnett’s test (**d**), and 2-way ANOVA with Sidak’s test (**e**). Significant interaction (P < 0.01) between treatment factors indicates that BCNU reduced hesperetin-induced stimulation ATP content.

Besides its prominent role in mitochondrial biogenesis, Pgc-1alpha is also involved in regulation of antioxidative function and suppresses reactive oxygen species at least in neurons^22^. This is partly mediated via increased expression of Nrf2 (nuclear factor erythroid 2 like 2, gene symbol Nfe2l2)^23^. Analysis of Nrf2 transcript levels indeed revealed that hesperetin treatment increased Nrf2 expression (Fig. 4a). While Pgc-1alpha is considered a master regulator of mitochondrial biogenesis, the transcription factor Nrf2 is playing a similar role in the control of cellular redox homeostasis by inducing a broad spectrum of genes that share an antioxidant response element (ARE) in their regulator region^24^. Therefore, we evaluated several known downstream targets of the Nrf2-ARE pathway involved in glutathione synthesis, ROS scavenging, xenobiotic metabolism, and drug elimination. Among those, two phase II detoxification enzymes (*Hmox1 and Nqo1*) and *Gclm*, the rate limiting enzyme of glutathione synthesis were significantly upregulated (Fig. 4b), while SOD and catalase expression was not changed (Suppl. Fig. 2). To verify the functional impact of hesperetin-induced Pgc-1alpha/Nrf2 signaling in primary differentiated myotubes we determined ROS and redox balance after challenge with the radical-forming tert-butyl hydroperoxide (TBHP). Indeed, hesperetin treatment reduced development of ROS measured by DHE live cell imaging (Fig. 4c). As Nrf2′s antioxidant function is mediated via regulation of glutathione production and regeneration, we determined reduced (GSH) and oxidized (GSSG) glutathione levels, one of the most important ROS scavengers. In accordance with our imaging experiments, hesperetin treatment increased the reduced/oxidized glutathione ratio (GSH/GSSG) emphasizing its ability to reduce oxidative stress (Fig. 4d). Nrf2 not only plays a prominent role in cellular redox homeostasis it recently emerged as multifaceted regulator of mitochondrial function and ATP production^24^. As hesperetin upregulated Gclm and improved the GSH/GSSG ratio, we wondered whether interference with glutathione biosynthesis could inhibit the metabolic effects of hesperetin. To answer this question we stimulated differentiated immortalized myotubes with hesperetin with or without the glutathione reductase inhibitor BCNU and analyzed our primary screening readout ATP content. The concomitant treatment of cells with BCNU significantly reduced hesperetin-induced increases in total ATP content (Fig. 4e) linking the antioxidant effects of the compound with its ability to improve mitochondrial energetics.

### Hesperetin treatment delays age-associated reduced performance and skeletal muscle atrophy in frail mice

**Figure 5:**
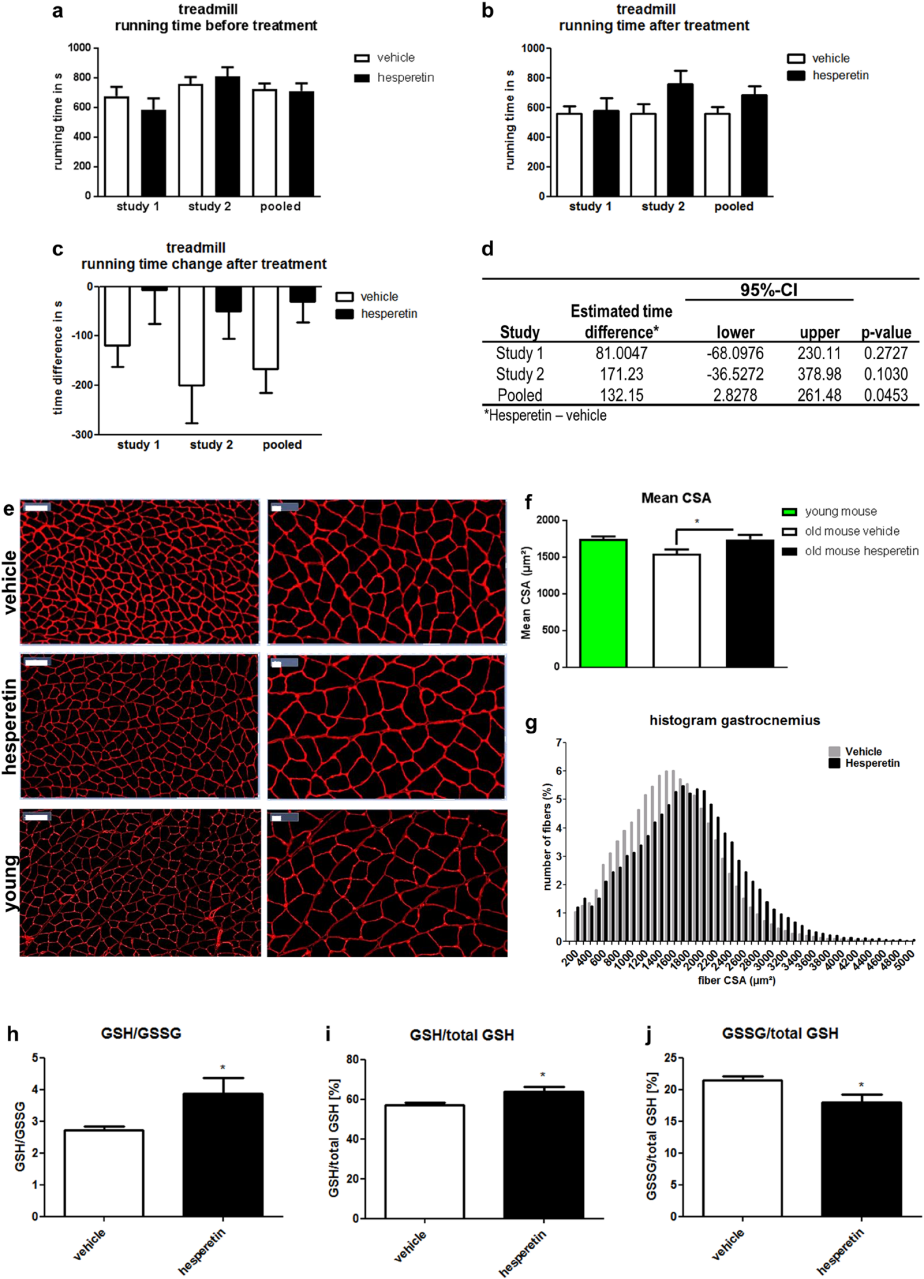
Hesperetin treatment of old mice improves endurance and delays skeletal muscle atrophy. (**a**–**d**) Analysis of voluntary treadmill running performance of old mice. Mice were analyzed before (**a**) and 6 weeks after treatment with 50 mg/kg hesperetin or vehicle (**b**). Individual and pooled results from two independent studies are shown (n ≥ 11 (1^st^ study), n ≥ 14 (2^nd^ study)). (**a**,**b**) Running time before and after treatment with 50 mg/kg hesperetin or vehicle (**c**) Running time change relative to baseline. (**d**) ANCOVA analysis of treatment effect on running time in treadmill. (**e**–**g**) Laminin staining of gastrocnemius from young and vehicle or hesperetin treated 23.5 month old mice. (**e**) Representative images. Scale bar left 100 µm, right 20 µm. **(f**) Comparison of gastrocnemius mean CSA in young (4 month, n = 6) and 23.5 month old mice treated with vehicle (n = 14) or hesperetin (n = 10). (**g**) Distribution of fiber CSA measured in laminin-stained gastrocnemius muscle. (**h**–**j**) GSH/GSSG (reduced/oxidized glutathione) content in quadriceps muscle from vehicle and hesperetin-treated old mice (n = 14 (vehicle), n = 11 (hesperetin)). The ratio of GSH/GSSG is a marker of oxidative stress. Reduced (GSH) and total glutathione (total GSH) were detected in separate experiments and GSSG was calculated from the total and reduced glutathione levels. Values represent means ± SEM. *P < 0.05; ***P < 0.001 ((**f**) One-Way ANOVA with Dunnett’s test; (**h**–**j**) two-tailed t-test).

The final goal of therapeutic intervention in sarcopenia is to improve physical performance of patients. Therefore, we decided to test hesperetin in aged (21.5 month old) mice using a treadmill endurance exercise protocol. Measuring running performance before and after treatment (Fig. 5a,b) showed that treadmill running time declined by about 100 s during the 8 week study period in vehicle-treated mice whereas hesperetin-treated animals maintained their running performance (Fig. 5c). However, despite testing more than 10 animals in each group changes in performance parameters did not reach significance (Fig. 5d). Nevertheless, analysis of muscle morphology and biochemical markers clearly indicated a response to compound treatment (see below). This prompted us to repeat the treadmill test with another even older batch of mice (23.5 months at baseline). Results of this study were consistent with the initial findings (Fig. 5c) and a pooled analysis of both experiments revealed that hesperetin treatment significantly delayed age-associated reduction of endurance in frail mice (Fig. 5d).

As activation of mitochondrial biogenesis by Pgc-1alpha is known to prevent denervation and fasting-induced loss of muscle mass^25^, we analyzed whether *in vivo* treatment with hesperetin, could also protect from skeletal muscle atrophy in our mouse model. Atrophy was evident in old vehicle-treated mice as shown by a reduced cross sectional area (CSA) of gastrocnemius muscle in comparison to young mice (Fig. 5e,f). Interestingly, CSA of hesperetin-treated mice was comparable to young mice (Fig. 5e,f) and significantly larger in comparison to the vehicle-treated controls (Fig. 5e–g). However, muscle hypertrophy was not uniformly observed in muscles of compound treated mice as whole muscle weight and lean mass were, despite a certain trend, not significantly increased (Suppl. Fig. 3a,b). Effects on fiber size seem to be independent of myogenic differentiation and regeneration as the levels of myogenin and embryonic myosin were not altered (Suppl. Fig. 3e). Analysis of molecular events that may underlie hesperetin’s muscle–protective effects largely confirmed our findings in myotubes. Similar to the *in vitro* experiments, hesperetin treatment slightly increased expression of citrate synthase, a marker of mitochondrial biogenesis. However, this upregulation did not result in increased enzyme activity precluding conclusions regarding the functional impact of this effect (Suppl. Fig. 3c,d). In contrast, oxidative stress in frail hesperetin-treated mice was noticeably reduced, suggesting that hesperetin’s antioxidant effects drive phenotypic improvements in our animal model (Fig. 5h–j). Taken together, these experiments indicate that boosting mitochondrial energy supply by hesperetin attenuated muscle atrophy and improved systemic muscle performance in aged mice.

## Discussion

Mitochondrial dysfunction and other age-related perturbations contributing to sarcopenia are highly complex and multifactorial conditions that are difficult to tackle using classical single target-based drug discovery strategies. Therefore, we analyzed here phenotypic readouts of mitochondrial bioenergetics - ATP content and respiration - to identify enhancers of mitochondrial energy production and muscle performance. Mitochondrial ATP production and oxidative capacity are validated measures of skeletal muscle function. Although the notion that oxidative capacity is generally decreased in aged muscle is controversial^26^, a number of studies suggest that mitochondrial dysfunction, ATP production, and decreased muscle quality correlate with low physical performance and mobility in older subjects^2–4^. We decided to test a natural product collection in this proof of concept study as a number of organic compounds are known as pleiotropic modulators of bioenergetic and metabolic pathways^27^. In keeping with this notion, our screening campaign resulted in the identification of 22 hits that significantly upregulated diverse functional measures of mitochondrial respiration *in vitro*.

Hesperetin emerged from screening as the most active compound in a series of flavanones, a compound class with a broad array of reported biological activities such as antioxidative and anti-inflammatory properties. Despite extensive studies in several cell types and organ systems^13,18^ there is sparse data on skeletal muscle specific effects of hesperetin. Jeong *et al*.^28^ showed that hesperetin improved injury-induced muscle regeneration *in vivo* via an enhancement of myogenic differentiation. Although we did not examine hesperetin’s effects on myogenic differentiation and regeneration in detail, myogenin and embryonic myosin transcript levels were unchanged after hesperetin treatment in frail mice. Furthermore, the role of satellite cells in the etiology of sarcopenia is – in contrast to acute muscle regeneration - highly debated^29^ making it less likely that hesperetin improved physical performance and prevented atrophy via enhancement of myogenic differentiation.

At the molecular level, hesperetin induced an upregulation of Pgc-1alpha and Nrf2 concomitant with increased antioxidant activity in differentiated myotubes. An increased GSH/GSSG ratio indicative of reduced oxidative stress was also seen in muscle of our hesperetin-treated mice. Both Pgc-1alpha and Nrf2 are known to increase mitochondrial respiration, spare capacity, ATP levels and mitochondrial oxidative phosphorylation complexes^20,24,30–33^, mimicking the cellular phenotype observed in our *in vitro* experiments. A very recent study identifying Nrf2 as novel target mediating hesperetin’s antioxidative effect in retinal pigment epithelial cells^34^ provides further evidence for engagement of the NRF axis by the compound. Interestingly, Pgc-1alpha and Nrf2 are both upregulated after exercise in humans^35,36^ and downregulated in sedentary old adults^2,37^. Moreover, Pgc-1alpha overexpression protects mice from development of skeletal muscle atrophy^25^ and reduces skeletal muscle fatigue^38^. On the other hand, Nrf2 knockout mice displayed reduced exercise-induced mitochondrial biogenesis, running capacity on treadmill^39^ and impaired skeletal muscle regeneration^40^. Again, these data are fully compatible with our observation that hesperetin treatment delays skeletal muscle atrophy in old mice and improves exercise performance.

Recently, the boundaries between Pgc-1alpha and Nrf2, originally seen as separate upstream regulators of either mitochondrial biogenesis or oxidative stress response, began to blur^22,24^. Importantly, the finding that Pgc-1alpha knockdown can inhibit Nrf2 induction after glutathione depletion^23^ suggests an intimate relationship between Pgc-1alpha and Nrf2 regulated antioxidant pathways. Vice versa, the effects of Nrf2 on Pgc-1alpha seem to be more complex and mainly apparent under stress conditions^24^ such as after S*taphylococcus aureus* induced lung injury where reduced induction of Pgc-1a in Nrf2 knockout mice was reported^41^. We show that hesperetin addresses this dual axis and that its metabolic benefits are dependent on the reduction of oxidative stress, however, the exact hierarchical relationship between Pgc-1alpha-driven and Nrf2-ARE-mediated effects remains to be elucidated.

In summary, the novel screening and profiling platform presented here enabled us to identify previously unknown effects of hesperetin on mitochondrial function in skeletal muscle. *In vitro* activity directly translated into therapeutic benefit in a complex physical function test in aged mice highlighting the value of the approach for identification of novel medicines targeting sarcopenia as well as other diseases connected with mitochondrial dysfunction.

## Methods

### Antibodies and reagents

Oligomycin, antimycin, FCCP, rotenone, AICAR, and Luperox (TBHP) were from Sigma. Antibodies against OXPHOS complexes (ab110413) and PGC-1alpha (ab106814) were from Abcam. Anti-GAPDH was from Cell Signaling (2118), anti-laminin from Dako (Z0097), anti-Tim23 (611223) and anti-RalA from BD (610221). Low glucose differentiation medium for immortalized human myoblasts was DMEM-F12 (US Biological) and for primary human skeletal muscle myoblasts (HSMM, Lonza) was DMEM (Gibco) supplemented with glucose (1 g/l) and 2% HS (Gibco). Galactose medium contained DMEM-F12 supplemented with galactose (1 g/l) and 2% HS.

### Screening and *in vitro* assays

Screening was performed with immortalized myotubes differentiated from a human skeletal myoblast line (hSkMc). HSkMc (kind gift from Dr. V. Mouly, Institut de Myologie, Université Pierre et Marie Curie) were derived from a 25 year old donor and immortalized by expression of human Tert and Cdk4 as described^42^. HSkMc were differentiated into myotubes in 96well format in low glucose medium for 5 days and afterwards stimulated with 10 µM compound for 24 h. In total 7949 pure natural products from the Sanofi compound collection were tested. Cellular ATP content was analyzed using Cell Titer Glo according to manufacturer’s instructions (Promega) with a Tecan Infinite M1000 reader (Tecan). Screening was performed in duplicates, whereby compounds were on two different plates with 100 µM AICAR as positive control. Compounds that increased ATP by more than 15% (>2 SD intra-plate of the DMSO negative control) were considered as hits. This setting allowed robust detection of our positive control that elevated ATP by 19.1–34.7% over all plates. In the secondary screen, mitochondria-dependent ATP content in differentiated immortalized human myotubes was analyzed after incubation with compounds (10 µM) for 21 h in low glucose medium followed by 3 h incubation in galactose medium with or without oligomycin. The difference between basal ATP content and ATP content in the presence of oligomycin was considered as mitochondrial ATP. Compounds that increased either total ATP or decreased glycolytic ATP significantly and increased mitochondrial ATP more than 7.5% (>1 SD intra-plate of the DMSO negative control) were considered as hits.

In all other experiments, primary myotubes were differentiated from HSMM (Lonza) and are described as primary differentiated myotubes. HSMM batches used in our experiments were from a 17 year old normal caucasian female with a BMI of 19 and isolated from upper arm or leg muscle tissue.

### Oxygen consumption in primary differentiated myotubes

Skeletal muscle metabolism of primary differentiated myotubes was analyzed using the Seahorse XFe 96 analyzer (Seahorse Bioscience). After 4 days of differentiation, myotubes were treated with 0.1% DMSO or compounds for 48 h. Afterwards mitochondrial respiration was measured with the XF Mito stress test kit in real-time according to the protocol provided by the manufacturer, whereby basal respiration, spare capacity, maximal respiration and ATP production were isolated by treatment of myotubes with 1.8 µM oligomycin, 1.1 µM FCCP and 1.1 µM antimycin/rotenone (see also Fig. 1f). Measurements were done in at least 8 replicates per plate and 1–5 independent experiments. Compounds that increased one or several of the analyzed respiratory parameters significantly were considered as hits. Spare capacity was defined as difference between maximal respiration (after FCCP injection) and basal respiration. Data were corrected to baseline (DMSO treated).

### *In vivo* experiments and physiological measurements

All animal care and experimental procedures were performed according to Sanofi Ethical Committee guidelines and to the Guide for the Care and Use of Laboratory Animals published by the National Institutes of Health. Sanofi is an authorized institution to house and handle laboratory animals according §11 German Animal Welfare Act. Procedures were approved by the competent authorities (Regierungspräsidium Darmstadt or Regierungspräsidium Giessen). 4 month old mice C57Bl6J mice were from Janvier labs. 21 or 23.5 month old C57Bl6J male mice (Janvier labs) were randomized in two groups based on their treadmill performance and treated for 8 weeks with 50 mg/kg hesperetin in 5% solutol (BASF) or vehicle (5% solutol) once daily p.o. Before and after 6 weeks of treatment treadmill performance of animals was analyzed under an endurance protocol on a Panlab 5-lane mouse treadmill (Harvard Apparatus, 5% incline). Mice were acclimatized to the treadmill by 2 days running without exhaustion. On the 3^rd^ day endurance was analyzed with the following protocol. During the first 2 minutes, the speed increased from 3 until 6 m/min. Thereafter speed increased continuously about 2 m/min every 2 min. Animals ran until exhaustion, which was defined as >3 falls/min. After 8 weeks, mice were sacrificed and skeletal muscles were dissected for histology, RNA, and protein analysis.

### RNA isolation and gene expression analysis

RNA was isolated from primary differentiated myotubes or tibialis anterior muscle from 23.5 month old male mice with the RNeasy mini kit according to manufacturer’s instructions (Qiagen). After reverse transcription, gene expression was analyzed with Taqman probes by the ViiA^TM^7 real-time PCR system (both Applied Biosciences). 18 s RNA was used as internal standard. MFC (microfluidic gene expression array cards) were customized for the following mitochondrial and myogenic genes: ACTB, GAPDH, RPL37A, TBP, ANKRD1, CPT1B, CS, FNDC5, FOXO1, FOXO3B;FOXO3, FST, HK2, IL15, IL15RA, IMMT, LRRK2, MSTN, MT-ATP6, MT-CYB, MT-ND2, MTOR, MYF5, MYH2, MYH4, MYH7, MYOG, NDUFA5, NOS3, NRF1, OPA1, PDK4, POLRMT, PPARD, PPARG, PPARGC1A, PPARGC1B, PRKAG2, PTGS1, RICTOR, SCD, SCO1, SDHD, TFAM, TFB1M, TIMP4, UCP3, VDAC1, VEGFA.

### Immunoblotting

After 4 days of differentiation, primary differentiated myotubes were stimulated with 0.1% DMSO or 10 µM hesperetin for 48 h. Afterwards cells were lysed, 10 µg lysate was separated by SDS-PAGE and incubated with different antibodies. Immunoreactive proteins were visualized on an Odyssey infrared imager (Li-Cor) and quantified with the Image Studio ver2.0 software (Li-Cor).

### Morphological Analysis

Gastrocnemius muscle from frail mice was embedded in Tissue-Tek (Sakura). Cryoblock was cut at thickest part of gastrocnemius and 10 µm thick sections were taken. Sections were fixed in acetone and stained with anti-Laminin antibody. Images were taken with an Axio Scan.Z1 microscope (Carl Zeiss). Cross-sectional area of myofibers from the whole cryosection (more than 5000 myofibers per section) was analyzed using custom-made software (Visiopharm).

### Oxidative stress and enzyme activity assays

After 4 days of differentiation in 96well format, primary differentiated myotubes were stimulated with 0.1% DMSO or 10 µM hesperetin for 48 h. Reduced (GSH) and oxidized (GSSG) glutathione was measured after 1 h induction of oxidative stress with 150 µM TBHP, and analyzed using GSH/GSSG-Glo assay according to the manufacturer’s instructions (Promega). To detect reactive oxygen species cells were washed with HBSS and incubated with 0.5 µM DHE for 10 minutes. Afterwards, DHE was removed and cells were stimulated with 100 µM TBHP for 30 minutes. Images of live cells were taken using ImageXPress MicroXL (Molecular Devices) and automatically analyzed (% area) using MetaXpress software. To analyze GSH and GSSG in quadriceps muscle of frail mice, skeletal muscle was snap frozen in liquid nitrogen and stored at −80 °C. To analyze the content of total and reduced glutathione, skeletal muscle was homogenized on dry ice in PBS/0.5% NP40 buffer, whereas buffer volume was adapted to the skeletal muscle weight. Reduced and total glutathione were detected in separate experiments using the same lysate and the GSH/GSSG ratio detection kit (Abcam ab138881). GSSG was calculated from the total and reduced glutathione levels.

### Statistical Analysis

Data are reported as mean ± SEM. Two groups were compared using unpaired t test, three or more groups using one-way ANOVA followed by Dunnett’s multiple comparison test. Combination treatments were analyzed by 2-way ANOVA followed by Sidak’s multiple comparison test. Values of p ≤ 0.05 were considered statistical significant. Screening results were automatically analyzed with a custom-made automated function written in the statistical programming language R (R Core Team (2015). R: A language and environment for statistical computing. R Foundation for Statistical Computing, Vienna, Austria) (generated by J. Ried). Treadmill data from two *in vivo* studies were analyzed with SAS 9.4. To adjust for baseline differences and to provide an unbiased estimate of the mean group effects an analysis of covariance (ANCOVA) was used. The linear model includes the factor treatment group, time at the end of the study and the time before start of treatment (baseline value) as a covariate.

## Electronic supplementary material

Supplementary Figures
