# Supplementary material for: High throughput screening of mitochondrial bioenergetics in human differentiated myotubes identifies novel enhancers of muscle performance in aged mice
Source: Sci Rep. 2018 Jun 20;8:9408. doi: 10.1038/s41598-018-27614-8 (PMC6010423; doi:10.1038/s41598-018-27614-8)
Supplement: Supplementary file 1 — Supplementary Figures [file 41598_2018_27614_MOESM1_ESM.pdf]

High throughput screening of mitochondrial bioenergetics in human differentiated myotubes identifies novel enhancers of muscle performance in aged mice

Nadine Biesemann[1*], Janina S. Ried[1], Danping Ding-Pfennigdorff[1], Axel Dietrich[2], Christine Rudolph[3], Steffen Hahn[4], Wolfgang Hennerici[4], Christian Asbrand[1], Thomas Leeuw[1] and Carsten Strübing[1*§]

Sanofi, Industriepark Hoechst, 65926 Frankfurt am Main, Germany
[1]R&D Immunology &Inflammation Therapeutic Area
[2]R&D TMED Bioinformatics
[3]R&D Integrated Drug Discovery
[4]R&D Clinical Science Operations

[*]Corresponding author, [§]Lead contact
e-mail: carsten.struebing@sanofi.com
e-mail: nadine.biesemann@sanofi.com

Supplementary Information

Supplementary Materials and Methods

Antibodies and reagents

Antibodies against OXPHOS complexes (ab110413) were from Abcam. Anti-GAPDH was from Cell Signaling (2118), MF20 from R&D (MAB4470), anti-RalA (610221) and anti-myogenin (556358) from BD. Low glucose medium for HSMM (human skeletal muscle myoblasts) was DMEM (Gibco) supplemented with glucose (1g/l) and 2% HS (Gibco). Primary myotubes were differentiated from HSMM (Lonza) and are described as primary differentiated myotubes. HSMM batches used in our experiments were from a 17 year old normal caucasian female with a BMI of 19 and isolated from upper arm or leg muscle tissue.

In vivo experiments and physiological measurements

All animal care and experimental procedures were performed according to Sanofi Ethical Committee guidelines and to the Guide for the Care and Use of Laboratory Animals published by the National Institutes of Health. Sanofi is an authorized institution to house and handle laboratory animals according §11 German Animal Welfare Act. Procedures were approved by the competent authorities (Regierungspräsidium Darmstadt or Regierungspräsidium Giessen). 21 month old C57Bl6J male mice (Janvier labs) were randomized in two groups based on their treadmill performance and treated for 8 weeks with 50mg/kg hesperetin in 5% solutol (BASF) or vehicle (5% solutol) once daily p.o. Before and after 4.5 weeks of treatment lean mass was using Minispec (Bruker). After 8 weeks, mice were sacrificed and skeletal muscles were dissected for histology, RNA, and protein analysis.

RNA isolation and gene expression analysis

After 4 days of differentiation, primary differentiated myotubes were stimulated with 10µM and 30µM hesperetin and corresponding DMSO concentrations for 48h and 72h. RNA was isolated either from primary differentiated myotubes or tibialis anterior muscle from 23.5month old male mice with the RNeasy mini kit according to manufacturer's instructions (Qiagen). After reverse transcription, gene expression was analyzed with Taqman probes by the ViiA^{TM}7 real-time PCR system (both Applied Biosciences). 18s RNA was used as internal standard.

Immunoblotting

After 4 days of differentiation, primary differentiated myotubes were stimulated with 0.1% DMSO or 10µM hesperetin for 48h. Afterwards cells were lysed, 10µg lysate was separated by SDS-PAGE and incubated with different antibodies. Immunoreactive proteins were visualized on an Odyssey infrared imager (Li-Cor) and quantified with the Image Studio ver2.0 software (Li-Cor).

Morphological Analysis

After 4 days of differentiation in 96well format, primary differentiated myotubes were stimulated with 0.1% DMSO or 10µM hesperetin for 48h. Myotubes were fixed with PFA and stained with antibodies against myogenin and DAPI and the corresponding secondary antibodies. 10x Images were taken with ImageXPress Micro XL (Molecular Devices) followed by automated analysis of 9 areas per well using MetaXpress software (Molecular Devices). Myogenin was analyzed by calculating the amount of Myogenin positive nuclei.

Enzyme activity assays

Citrate synthase activity was determined in quadriceps muscles according to manufacturer's instructions (Sigma CS0720).

Statistical Analysis

Data are reported as mean ±SEM. Two groups were compared using unpaired t test, three or more groups using one-way ANOVA followed by Dunnett's multiple comparison test. Values of $p \leq 0.05$ were considered statistical significant.

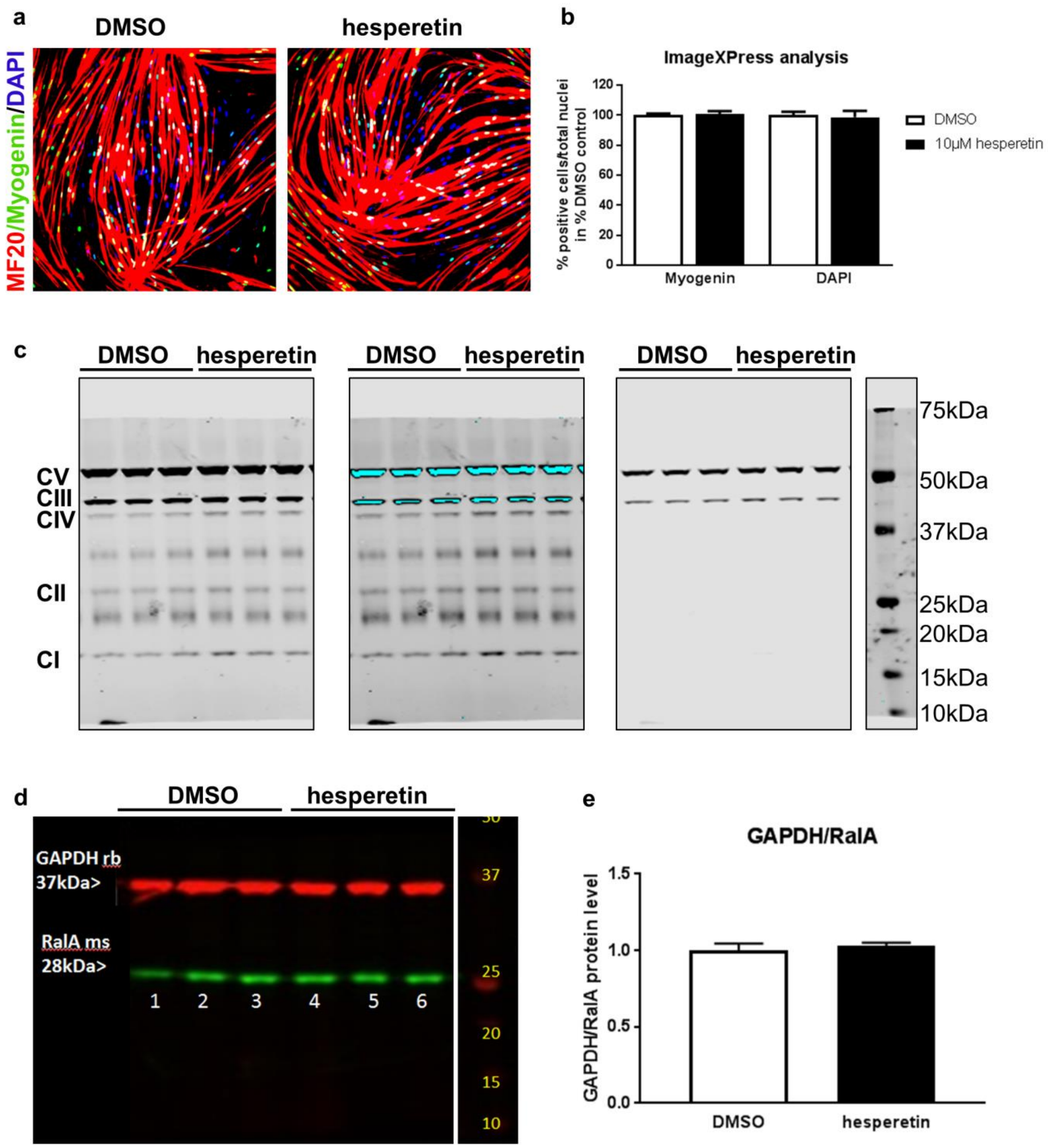

Suppl. Fig. 1 Hesperetin induces mitochondrial biogenesis

(a-e) Primary differentiated myotubes were treated 48h with 10µM hesperetin or DMSO. (a-b) Myotubes were stained with anti-myogenin and DAPI and analyzed with ImageXPress (9 areas per well, n= 59 (DMSO), n= 20 (hesperetin), 3 independent experiments). (c) Western Blot analysis of respiratory complexes. Due to higher protein levels of complex V and III (compared to respiratory complexes I, II and IV), exposure time was adapted (left: medium, middle: high, right: low exposure time). Blue colour indicates overexposed bands. Middle and right blots are cropped and shown in Fig. 3c. Gels were cropped below 75kDa. (d-e) Western Blot analysis of two loading controls GAPDH and RalA (n=3). Gels were cropped below 50kDa. Values represent means ±SEM.

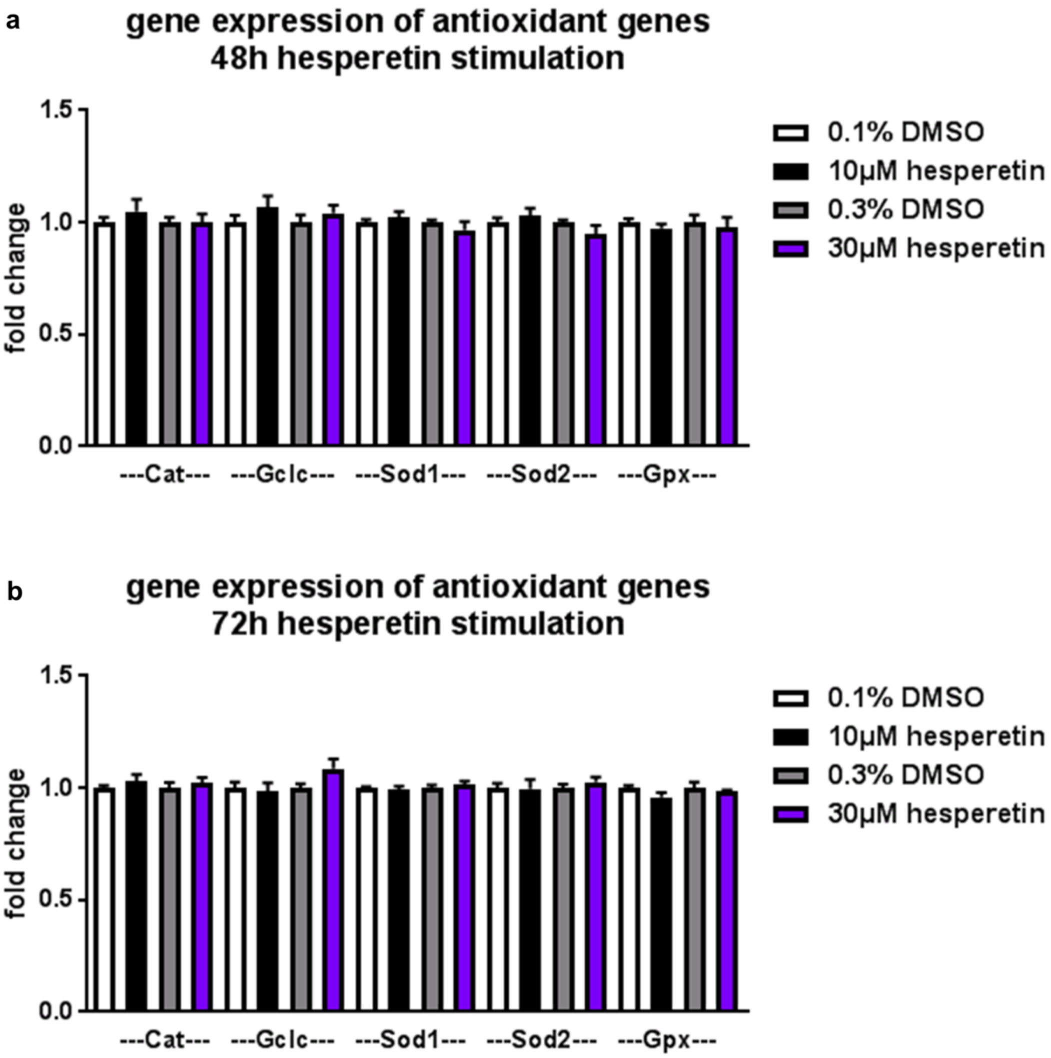

Suppl. Fig. 2 Hesperetin is not inducing catalase and Sod gene expression

Human primary differentiated myotubes were treated with hesperetin or DMSO. (a-b) Taqman analysis of Nrf2-ARE target genes after 48h (a) or 72h (b) stimulation with hesperetin (n=6). Values represent means ±SEM.

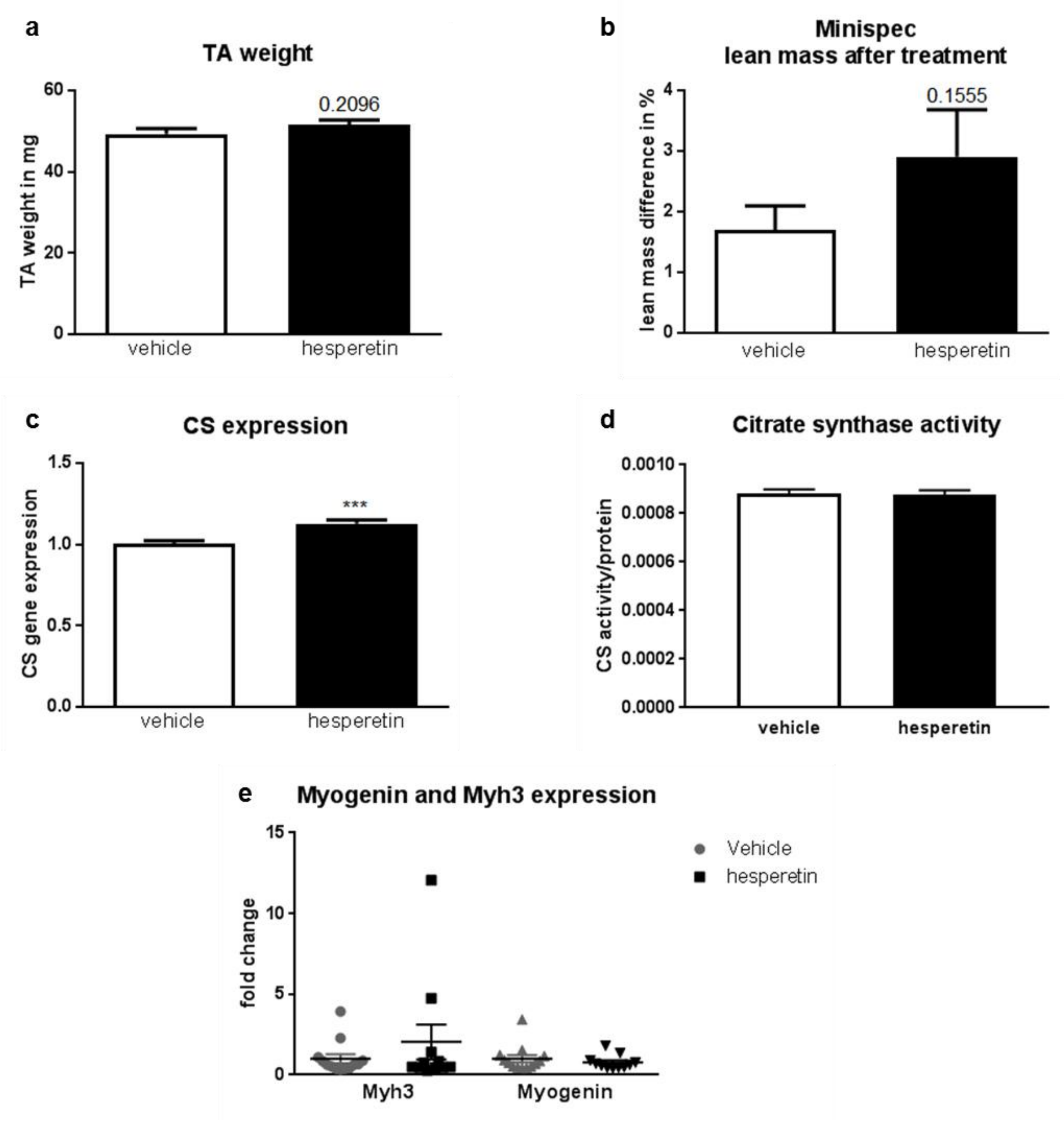

Suppl. Fig. 3 Hesperetin tends to increase skeletal muscle weight and lean mass in frail mice

21month old mice were treated for 8 weeks with 50mg/kg hesperetin (n=11) or vehicle (n=14). (a) Skeletal muscle weight of tibialis anterior. (b) Minispec MRI analysis of lean mass from vehicle and hesperetin-treated old mice after 4.5 weeks treatment. Shown is lean mass difference in % of baseline measurements. (c) Taqman analysis of citrate synthase expression in TA muscle from vehicle and hesperetin-treated old mice. (d) Citrate synthase activity in quadriceps muscle from vehicle and hesperetin-treated old mice. (e) Taqman analysis of Myh3 and myogenin expression in TA muscle from vehicle and hesperetin-treated old mice. Values represent means ±SEM. *** $P<0.001$ (two-tailed t-test).
